# Metabolic syndrome in patients with schizophrenia: Why should we care

**DOI:** 10.1097/MD.0000000000029775

**Published:** 2022-08-12

**Authors:** Jichao Liu, Lijuan Fu

**Affiliations:** a Department of Psychiatry, Tianjin Anding Hospital, Hexi District, Tianjin, China.

**Keywords:** care, factors, management, metabolic syndrome, schizophrenia

## Abstract

Metabolic syndrome (MS) is a serious disease in patients with schizophrenia; it is necessary to evaluate the characteristics and influencing factors of MS to provide reliable evidence for the management of schizophrenia.

Patients with schizophrenia treated in our hospital from January 1, 2018, to March 31, 2021, were selected. The characteristics and treatment details of MS and no-MS patients were evaluated. Pearson correlation analyses were applied for analyzing MS and related characteristics. Logistic regression analyses were conducted to evaluate the risk factors of MS in patients with schizophrenia.

A total of 465 patients with schizophrenia were included, the incidence of MS in patients with schizophrenia was 18.06%. Pearson correlation analyses had found that age (r = 0.621), waist circumference (r = 0.744), body mass index (r = 0.691), diabetes (r = 0.598), course of disease (r = 0.504), triglyceride (r = 0.532), high-density lipoprotein cholesterol (r = –0.518), low-density lipoprotein cholesterol (r = 0.447), and total cholesterol (r = 0.523) were correlated with MS (all *P* < .05). Logistic regression analyses showed that age ≥55 years (odds ratio [OR]: 2.012, 95% confidence interval [CI]: 1.425–3.196), waist circumference ≥80 cm (OR: 1.944, 95% CI: 1.081–3.172), body mass index ≥24.5 kg/m^2^ (OR: 2.451, 95% CI: 1.825–3.108), diabetes (OR: 2.301, 95% CI: 1.944–2.881), course of disease ≥15 years (OR: 1.804, 95% CI: 1.236–2.845), triglyceride ≥1.5 mmol/L (OR: 2.032, 95% CI: 1.614–3.079), high-density lipoprotein cholesterol ≤0.8 mmol/L (OR: 1.226, 95% CI: 1.102–1.845), low-density lipoprotein cholesterol ≥2 mmol/L (OR: 1.759, 95% CI: 1.236–1.987), and total cholesterol ≥4.5 mmol/L (OR: 1.664, 95% CI: 1.422–1.852) were the risk factors of MS in patients with schizophrenia (all *P* < .05).

MS is very common in patients with schizophrenia, which may be associated with many possible risk factors, and early interventions and nursing care targeted at those influencing factors are needed to improve the prognosis of schizophrenia.

## 1. Introduction

Schizophrenia is a brain disease characterized mainly by long-term persistence or continuous aggravation of a variety of mental symptoms, although there are cases with better prognosis which are treatable.^[1,2]^ Compared with the general population, patients with schizophrenia have a higher risk of all-cause mortality, and a shortened life expectancy of about 20%.^[3]^ Approximately 60% of death of patients with mental illness has been observed to be related to cardiovascular disease.^[4]^ Therefore, the management of cardiovascular disease is essential to the prognosis of schizophrenia.

With the development of economy and medical science, the prognosis of patients with schizophrenia has undergone significant changes, such as increased treatment and remission rates, improved compliance, reduced serious adverse drug reactions, and improved quality of life.^[5–7]^ At the same time, due to the application of antipsychotics, long-term hospitalization, or poor life and eating habits, a metabolic syndrome (MS) that integrates various metabolic risk factors such as obesity, abnormal glucose metabolism, dyslipidemia, and hypertension has gradually become a prominent problem of schizophrenia patients.^[8,9]^ In China, schizophrenia is generally diagnosed with the tool of Chinese classification of mental disorder (CCMD); it has been reported^[10,11]^ that CCMD have similar diagnostic credibility of schizophrenia to an international classification system such as the International Classification of Diseases or Diagnostic and Statistical Manual of Mental Disorders. The prevalence of schizophrenia in European and American countries is 30.14% to 50.09%, and the prevalence in Asian countries is about 15.57% to 40.24%, which is 2 to 4 times higher than that of general population.^[12,13]^ A previous study^[14]^ has pointed out that patients with schizophrenia have a higher prevalence of MS compared with no schizophrenia controls (20.11% vs 5.45%). The related influencing factors of the high MS prevalence in schizophrenia are still unclear. Therefore, this study analyzed the clinical characteristics of patients with schizophrenia admitted to our hospital to identify the predictors of MS in patients with schizophrenia and to explore relevant countermeasures for the prevention and treatments of MS.

## 2. Methods

### 2.1. Ethical approval

In this study, all methods were performed in accordance with the Strengthening the Reporting of Observational Studies in Epidemiology statement. This present study obtained the ethical approval from the ethics committee of Tianjin Anding Hospital with approval number: 180064. Written informed consents had been obtained from all the legal guardians (the parents, the sons, or daughters) of included patients with schizophrenia.

### 2.2. Patients

We selected patients with schizophrenia who were treated in our hospital from January 1, 2018, to March 31, 2021, as the research population. The inclusion criteria for patients were: age ≥18 years; disease diagnosis met the Chinese classification of mental disorder (CCMD) version 3 diagnostic criteria for schizophrenia; patients accepted treatment in our hospital; the guardians of included patients had been well informed and signed the written informed consents. The patients were excluded if the medical records were incomplete and the guardians or patients did not agree to participate in this study.

### 2.3. MS diagnosis

The diagnosis of MS adopts the diagnostic criteria recommended by the International Diabetes Federation^[15]^: central obesity (waist circumference ≥90 cm in men, ≥80 cm in women), combined with any 2 of the following 4 indicators: elevated triglycerides (TG >1. 7 mmol/L) or have received corresponding treatment; high-density lipoprotein cholesterol (HDL-C) is lowered (male HDL-C <0.9 mmol/L or female <1.1 mmol/L); blood pressure is increased (systolic blood pressure ≥140 or disystolic blood pressure ≥90 mm Hg); fasting blood glucose is elevated (≥5.6 mmol/L).

### 2.4. Data collection

Two investigators independent evaluated and collected the following data and information: age, gender, waist circumference, body mass index (BMI), alcohol drinking, smoking, hypertension, diabetes, drug treatment, course of disease, and results of laboratory examinations including TG, HDL-C, low-density lipoprotein cholesterol (LDL-C), total cholesterol, apolipoprotein A1, apolipoprotein B, C-reactive protein, uric acid, creatinine, urea nitrogen, total protein, albumin, and globulin.

### 2.5. Statistical analysis

We used SPSS 23.00 statistical software to process the collected data. Measurement data were expressed as mean ± standard deviation; independent sample *t* test was used for comparison between groups; count data were expressed as percentage (%), and chi-square test was used for comparison between groups. Pearson correlation analyses were applied for analyzing MS and related characteristics. Logistic regression analyses were conducted to evaluate the risk factors of MS in patients with schizophrenia. In this study, a *P* value of <.05 was considered as the difference between the groups was statistically significant.

## 3. Results

### 3.1. Characteristics of included patients

A total of 465 patients with schizophrenia were included in this study, of whom 84 patients had been diagnosed with MS; the incidence of MS in patients with schizophrenia was 18.06%. As shown in Table 1, there were significant differences in age, waist circumference, BMI, diabetes, course of disease, TG, HDL-C, LDL-C, and total cholesterol (all *P* < .05). There were no significant differences in the gender, alcohol drinking, smoking, hypertension, drug treatment, apolipoprotein A1, apolipoprotein B, C-reactive protein, uric acid, creatinine, urea nitrogen, total protein, albumin, and globulin between MS and no-MS groups (all *P* > .05).

**Table 1:** The characteristics of included patients.

Variables	MS group (n = 84)	No-MS group (n = 381)	t/χ^2^	*P* value
Age (y)	61.24 ± 7.04	50.63 ± 8.27	1.531	0.087
Male/female	45/39	204/177	2.206	0.124
Waist circumference (cm)	86.81 ± 20.14	75.44 ± 21.09	7.227	0.009
BMI (kg/m^2^)	27.13 ± 1.29	22.62 ± 1.77	5.143	0.031
Alcohol drinking	36 (42.86%)	160 (41.99%)	1.322	0.065
Smoking	29 (34.52%)	128 (33.59%)	1.425	0.103
Hypertension	55 (65.48%)	211 (55.38%)	1.813	0.052
Diabetes	62 (73.81%)	90 (23.62%)	1.885	0.011
Drug treatment			1.063	0.089
Clozapine	10 (11.90%)	62 (16.27%)		
Chlorpromazine	24 (28.57%)	94 (24.67%)		
Olanzapine	10 (11.90%)	48 (12.59%)		
Risperidone	29 (34.52%)	126 (33.07%)		
Quetiapine	6 (7.14%)	26 (6.82%)		
Other	5 (5.95%)	25 (6.56%)		
Course of disease (y)	21.56 ± 5.31	12.01 ± 6.18	3.024	0.005
Triglyceride (mmol/L)	2.19 ± 1.14	1.12 ± 0.87	1.146	0.013
HDL-C (mmol/L)	0.62 ± 0.24	1.04 ± 0.88	1.232	0.037
LDL-C (mmol/L)	2.68 ± 1.01	1.45 ± 0.96	1.055	0.016
Total cholesterol (mmol/L)	4.99 ± 1.76	4.13 ± 1.27	2.384	0.047
Apolipoprotein A1 (g/L)	0.98 ± 0.33	0.95 ± 0.29	1.131	0.112
Apolipoprotein B (g/L)	0.78 ± 0.27	0.61 ± 0.24	1.659	0.042
C-reactive protein (mg/L)	3.52 ± 2.09	3.24 ± 2.11	1.883	0.057
Uric acid (μmol/L)	4.31 ± 1.76	4.28 ± 1.66	2.005	0.108
Creatinine (μmol/L)	74.97 ± 21.04	72.78 ± 19.41	16.429	0.156
Urea nitrogen (mmol/L)	4.31 ± 1.68	4.32 ± 1.83	2.231	0.084
Total protein (g/L)	78.11 ± 27.43	76.38 ± 22.49	9.114	0.055
Albumin (g/L)	45.06 ± 8.13	44.12 ± 9.34	11.286	0.112
Globulin (g/L)	26.15 ± 11.27	26.35 ± 13.09	7.129	0.081

### 3.2. Correlation of MS and related characteristics

As indicated in Table 2, Pearson correlation analyses had found that age (r = 0.621), waist circumference (r = 0.744), BMI (r = 0.691), diabetes (r = 0.598), course of disease (r = 0.504), TG (r = 0.532), HDL-C (r = –0.518), LDL-C (r = 0.447), and total cholesterol (r = 0.523) were correlated with MS (all *P* < .05).

**Table 2:** Pearson correlation analysis of MS and related characteristics.

Variables	r	*P* value
Age (y)	0.621	.008
Gender	0.019	.125
Waist circumference (cm)	0.744	.013
BMI (kg/m^2^)	0.691	.022
Alcohol drinking	0.115	.081
Smoking	0.074	.101
Hypertension	0.105	.072
Diabetes	0.598	.029
Drug treatment	0.078	.107
Course of disease (y)	0.504	.042
Triglyceride (mmol/L)	0.532	.015
HDL-C (mmol/L)	-0.518	.032
LDL-C (mmol/L)	0.447	.018
Total cholesterol (mmol/L)	0.523	.025
Apolipoprotein A1 (g/L)	0.114	.109
Apolipoprotein B (g/L)	0.157	.087
C-reactive protein (mg/L)	0.092	.101
Uric acid (μmol/L)	0.178	.113
Creatinine (μmol/L)	0.024	.125
Urea nitrogen (mmol/L)	0.118	.099
Total protein (g/L)	0.093	.106
Albumin (g/L)	0.044	.158
Globulin (g/L)	0.106	.074

### 3.3. Risk factors of MS

The variable assignments of multivariate logistic regression are presented in Table 3. Logistic regression analyses showed that age ≥55 years (OR: 2.012, 95% CI: 1.425–3.196), waist circumference ≥80 cm (OR: 1.944, 95% CI: 1.081–3.172), BMI ≥24.5 kg/m^2^ (OR: 2.451, 95% CI: 1.825–3.108), diabetes (OR: 2.301, 95% CI: 1.944–2.881), course of disease ≥15 years (OR: 1.804, 95% CI: 1.236–2.845), TG ≥1.5 mmol/L (OR: 2.032, 95% CI: 1.614–3.079), HDL-C ≤ 0.8 mmol/L (OR: 1.226, 95% CI: 1.102–1.845), LDL-C ≥2 mmol/L (OR: 1.759, 95% CI: 1.236–1.987), and total cholesterol ≥4.5 mmol/L (OR: 1.664, 95% CI: 1.422–1.852) were the risk factors of MS in patients with schizophrenia (all *P* < .05).

**Table 3:** The variable assignments of multivariate logistic regression.

Factors	Variables	Assignment
MS	Y	yes = 1, no = 2
Age (y)	X_1_	≥55 = 1, <55 = 2
Waist circumference (cm)	X_2_	≥90 = 1, <90 = 2
BMI (kg/m^2^)	X_3_	≥26 = 1, <26 = 2
Diabetes	X_4_	Yes = 1, no = 2
Course of disease (y)	X_5_	≥15 = 1, <15 = 2
Triglyceride (mmol/L)	X_6_	≥1.5 = 1, 1.5 = 2
HDL-C (mmol/L)	X_7_	≤0.8 = 1, >0.8 = 2
LDL-C (mmol/L)	X_8_	≥2 = 1, <1 = 2
Total cholesterol (mmol/L)	X_9_	≥4.5 = 1, <4.5 = 2

## 4. Discussions

The problem of MS associated with mental illness especially schizophrenia is receiving more and more attention from researchers and clinicians. Different countries or organizations have proposed MS diagnostic standards, and the degree of leniency and strictness of these standards varies greatly.^[16]^ Therefore, the prevalence of MS in patients with schizophrenia varies greatly in different countries and regions. Several studies^[17–19]^ have shown that the prevalence of MS in schizophrenia is higher than that of the general population. This study shows that the prevalence of MS in schizophrenia is 18.06%, which is significantly >14.10% of the general population in Shanghai. Besides, we have found that age, waist circumference, BMI, diabetes, course of disease, TG, HDL-C, LDL-C, and total cholesterol are correlated with MS; early alertness and more attention are needed for those patients.

The metabolic disorders of schizophrenia are multifaceted, including disorders of sugar, lipid, and protein metabolism.^[20]^ Previous studies^[21–23]^ have found that the abnormal rates of waist circumference and HDL-C in patients with schizophrenia are 70% and 61%, respectively, and those with abnormalities in 1 to 4 indicators are 20%, 28%, 34%, and 10%, respectively. The prevalence of MS in the schizophrenia group (28.4%) is higher than that of no schizophrenia group (3.3%), specifically the prevalence of female patients (39.8%) is higher than that of male patients (22.4%), and the prevalence of patients over the age of 39 years (30.9.3%) is higher than that of patients with age <39 years (22.1%).^[24]^ Sugawara et al^[25]^ report on the MS prevalence of Japanese schizophrenia patients (27.5%), which is lower than that reported in the United States and Canada (43% and 46%).^[26,27]^ These results suggest that metabolic abnormalities in patients with schizophrenia are very common.^[28]^ When 1 or 2 indicators are abnormal, attention should be paid to the timely adjustment of the treatment plan or early preventive interventions.

The prevalence of MS in patients with schizophrenia is significantly higher than that of the general population.^[29]^ It has been reported that in addition to general risk factors such as advanced age, heredity, and bad living habits, patients with schizophrenia also have risk factors closely related to the disease, such as drugs use.^[30]^ This study has found that dyslipidemia (increased TG and decreased HDL-C), obesity (especially central obesity), and increased blood sugar are the most important risk factors. However, the mechanism of these risk factors leading to MS is currently not fully understood. Many studies^[31,32]^ suggest that changes in dopamine and serotonin neurotransmitters and receptors caused by schizophrenia or psychotropic drugs and changes in the expression levels of certain genes may be the underlying pathogenesis. Although the mechanism of these risk factors leading to MS is still unclear, understanding those risk factors has important clinical significance for the prevention and treatment of MS. The main drugs used in the schizophrenia patients in this study were the chlorpromazine, olanzapine, and risperidone. Such antipsychotic drugs have a very wide range of applications in psychiatry. They can effectively block D2 receptors and 5-HT2A and have ideal antihallucination and antidelusional effects. However, some patients are at risk of developing MS after taking medication, and the current clinical mechanism for causing MS is still inconclusive, which may be related to the blockade effects of monoamine neurotransmitters such as histamine, norepinephrine, M receptors, and 5-HT.^[33–35]^ After the monoamine neurotransmitter is blocked, the patient’s appetite is enhanced, and it also has a sedative effect, which can increase the patient’s food intake, reduce the amount of exercise, and increase the accumulated energy in the body. It is easy to induce symptoms such as insulin resistance and increased BMI, leading to abnormal glucose and lipid metabolism in patients with schizophrenia. A previous study^[36]^ has pointed out that about 50% of patients who take antipsychotic drugs for 6 months may have symptoms such as weight gain or obesity, which will have a certain impact on their treatment compliance, so it is difficult to ensure the stability of mental illness treatment. Therefore, the impact of clinical drug use on MS is worthy of further research in the future.

Previous research results^[37,38]^ have shown that drug intervention can alleviate patients’ metabolic problems, but due to drug side effects, nondrug interventions such as behavioral and nutritional therapy may be the first choice for intervention. Studies^[39–41]^ have shown that exercise, diet, and education interventions can help reduce and control weight gain. Lifestyle interventions are safer and more effective in reducing or maintaining weight and can improve the quality of life. Studies^[42–44]^ have found that moderate-intensity exercise may be beneficial to improve the positive and negative symptoms and cognitive function of patients with schizophrenia and reduce the risk factors of various common health problems, including MS and tobacco and substance use. Although studies^[45,46]^ have shown that doctors’ weight loss recommendations will generally affect patients’ weight loss behavior and actual weight loss, the doctor’s recommendations may not be strong enough to affect weight and metabolic abnormalities. Besides, the cognitive dysfunction of patients with psychiatric disorders limits their understanding of the basic advice provided by psychiatrists. Therefore, future studies on the effects and safety of MS intervention and nursing care are needed in patients with schizophrenia.

There are some deficiencies in this study worth considering. First, this study is a single-center observational study with limited sample size. Second, the relevant influencing factors included in this study are limited, and there may be other relevant MS influencing factors that we did not include in the analysis. The risk factors of MS need to be further confirmed by studies with a wider range and larger sample size. Furthermore, more studies are warranted to investigate the preventative and coping measures of MS in patients with schizophrenia, to improve the prognosis of patients with schizophrenia.

## 5. Conclusions

In summary, MS is very common in patients with schizophrenia. For schizophrenia patients with age ≥55 years, waist circumference ≥80 cm, BMI ≥24.5 kg/m^2^, diabetes, course of disease ≥15 years, TG ≥1.5 mmol/L, HDL-C ≤0.8 mmol/L, LDL-C ≥2 mmol/L, and total cholesterol ≥4.5 mmol/L, they may have higher risks of MS; early alertness and special attention are needed for patients with those factors to reduce the MS in patients with schizophrenia.

**Table 4:** Logistic regression analysis on the risk factors of MS.

Variables	β	Wald	OR	95% CI	*P* value
Age ≥55 y	0.118	0.124	2.012	1.425–3.196	.013
Waist circumference ≥80 cm	0.125	0.101	1.944	1.081–3.172	.034
BMI ≥24.5 kg/m^2^	0.103	0.115	2.451	1.825–3.108	.024
Diabetes	0.114	0.127	2.301	1.944–2.881	.017
Course of disease ≥15 y	0.187	0.129	1.804	1.236–2.845	.047
Triglyceride ≥1.5 mmol/L	0.104	0.164	2.032	1.614–3.079	.011
HDL-C ≤0.8 mmol/L	0.126	0.142	1.226	1.102–1.845	.028
LDL-C ≥2 mmol/L	0.115	0.158	1.759	1.236–1.987	.033
Total cholesterol ≥4.5 mmol/L	0.121	0.173	1.664	1.422–1.852	.016

## Author contributions

Lijuan Fu designed research, wrote the first draft of article, and had primary responsibility for final content. Jichao Liu and Lijuan Fu conducted research and analyzed data. All authors read and approved the final article.
